# The effect of media golf professionals’ image on instructor trust and customer satisfaction

**DOI:** 10.3389/fpsyg.2025.1656378

**Published:** 2025-10-01

**Authors:** Ga-Young Kim, Youbin Kim, Hye Jin Yang, Chul-Ho Bum, Chulhwan Choi

**Affiliations:** ^1^Department of Physical Education, Graduate School, Kyung Hee University, Yongin-si, Republic of Korea; ^2^College of Physical Education, Kyung Hee University, Yongin-si, Republic of Korea; ^3^Department of Physical Education, Gachon University, Seongnam-si, Republic of Korea

**Keywords:** sport media, golf professional, instructor trust, customer satisfaction, image

## Abstract

Since media golf professionals are mainly encountered online rather than through face-to-face instruction, it is difficult to assess their competency and expertise as instructors. To this end, the study sought to assess instructors’ instructional competence through the construct of instructor image and examine its influence on instructor trust and customer satisfaction. The participants were 290 Korean adult amateur golfers (186 men and 104 women). A questionnaire was used to measure perceptions of instructor image, instructor trust, and customer satisfaction. Data were analyzed using descriptive statistics, validity and reliability tests, and multiple regression analysis. First, the instructor image of media golf professionals was found to have a partially positive effect on instructor trust. Among the subfactors of instructor image, attitude did not have a significant effect. Second, instructor image had a significantly positive effect on customer satisfaction; however, among its sub-factors, qualification did not have a significant impact. Third, instructor trust had a significantly positive effect on customer satisfaction. In conclusion, the findings of this study state that a positive instructor image of media-based golf professionals fosters trust and satisfaction, thereby promoting continued participation in exercises and sustained viewing intentions.

## 1 Introduction

With the advancement of digital technology, people can access a wide range of online content without the limitations of time and place (Knell, 2021). The golf industry has shown a growing trend in content distribution on online platforms. Instagram is a representative platform for sharing photos and short videos (Hu et al., 2014), whereas YouTube and Netflix are major video platforms. With the widespread use of mobile devices and tablet PCs, the use of OTT services has also expanded. Unlike traditional TV broadcasting, online platforms allow users to watch videos and obtain information conveniently (Puthiyakath and Goswami, 2021). These platforms enable individuals to promote themselves, share content, and acquire new knowledge through social networking sites (SNS) and other online media.

In the past, golf was widely perceived as an expensive sport that was difficult to access. However, with the expansion of screen golf, a more affordable alternative, golf has become more accessible and popular among the general public (Lee and Kwon, 2021). As the number of golf participants has increased, online lesson content on YouTube and golf broadcasts have become more widespread and frequently consumed (Sorbie et al., 2020). Previously, golfers had to physically visit a location to receive lessons but the rise in online lesson content has allowed easier access to information. This trend has also provided an opportunity to effectively convey information online, allowing viewers and presenters to communicate and interact through dialogic content in digital environments (Zhang et al., 2024). As a result, people have increasingly turned to video-based content when they seek to learn something (Rosenthal, 2018; Jebe and Park, 2019).

Viewers often feel a sense of familiarity and closeness with the presenter, even without having met them in person (Kowert and Daniel, 2021). This phenomenon where individuals engage and form a sense of relationship with a figure beyond the screen is referred to as parasocial interaction (PSI) or a parasocial relationship (PSR) (Horton and Richard Wohl, 1956; Rubin et al., 1985; Frederick et al., 2012). These experiences can arise from various factors, such as whether the instructor possesses a likable or charismatic personality (Rubin and McHugh, 1987; Lee and Watkinson, 2016), and whether they consistently exhibit the same attitudes and beliefs over time (Turner, 1993; Frederick et al., 2012). To minimize the perceived disconnection between online and offline environments, it is important for the instructor to convey a sense of warmth and closeness to the viewer (Beautemps and Bresges, 2021). Moreover, transitioning from relatively short-term PSI to a more sustained and enduring PSR (Liebers and Schramm, 2019) requires the presenter to not only demonstrate subject-matter expertise but also possess the ability to evoke empathy from learners.

Instagram has also gained recognition as an effective promotional tool across various fields, increasing the influence of social media influencers (Brag, 2019). Many golf professionals now produce and share short-form content, such as brief lesson clips or swing videos, on Instagram (Lee and Kim, 2024). When individuals present themselves through social networking services (SNS), they can imprint their self-image on learners and viewers while also creating a foundation for the development of parasocial interactions (Kim and Song, 2016). Moreover, platforms such as YouTube and Instagram allow viewers to provide immediate responses and feedback, and when viewers observe that their comments or suggestions are acknowledged and reflected, their interest and engagement tend to increase (Bond, 2016). As the influence of the online market continues to grow (Haenlein et al., 2020), golf instructors must strategically utilize online platforms to promote themselves and maintain competitiveness. Additionally, media professionals actively appear in videos of popular YouTubers or participate in popular golf tournaments, such as the G-Tour, to gain media exposure (Kang and Park, 2015). As online content delivers information easily and offers entertainment value (Wendt et al., 2016), golf instructors who appear in such content can build public recognition more effectively.

Previously, golf professionals were categorized as teaching professionals, who primarily focus on instruction, and tour professionals, who compete in tournaments. With the development of media, a new group of professionals has emerged, referred to as media professionals, who are primarily active in broadcasting or social media. These individuals often attract attention owing to their attractive appearance or favorable physical attributes and, like tour players, receive sponsorship for clothing and equipment. The widespread use of sponsorship in sports reflects its strong marketing impact on athletes and influencers (Erdogan et al., 2001).

Previously, sports instructors were evaluated primarily based on their teaching abilities. However, Housner and French (1994) stated that sports instructors should be evaluated not only for their teaching ability but also for their skills in sports and their knowledge of sports education. In addition, golf instructors are expected to cultivate a positive mindset toward the sport, beyond just teaching technical skills (Cromack, 2011). In contrast to the previously performance-oriented perception of athletes, increasing visibility through media has shifted the criteria for determining their value, with not only performance but also appearance and image playing significant roles (Wolter, 2010). Therefore, athletes must use online media as an essential tool for self-promotion (Davies and Mudrick, 2017).

This study aims to examine how amateur golfers perceive the instructor image of media golf professionals through broadcasts and online platforms, and how these perceptions influence instructor trust and customer satisfaction. Boulding et al. (1964) defined image as the mental picture of an individual based on subjective perceptions. An instructor’s image is derived from their competency, expertise, role, and attitude, which together foster psychological stability and build trust in the instructor (Kolter and Clarke, 1987). Kumar (1996) described trust as a fundamental concept for forming positive relationships and defined it as the emotional expectation that the other party will meet one’s needs. The confidence inspired by the instructor has a significant effect on learners’ psychological stability (Cook and Wall, 1980). In the case of media-based instruction, learners receive lessons from media professionals’ functions not only as students but also as customers. Their satisfaction is crucial for maintaining long-term golf participation and continued engagement with lesson content. Satisfaction is an emotional response based on personal experience and performance (Tiffin and McCormick, 1965). Customer satisfaction, specifically, refers to emotional reactions to the quality of services received (Howard and Sheth, 1969). Thus, it is not simply the instructor’s appearance but the quality of the lesson content and its impact on the learner’s performance that influences satisfaction. Because media professionals play the dual roles of instructors and content providers, it is important to examine their effectiveness using these constructs.

An effective instructor must possess not only advanced instructional skills (Mageau and Vallerand, 2003) but also professional knowledge that supports learners’ development (Sucipto et al., 2017). These qualities help foster trust in the instructor, which is essential for learner satisfaction, and play a critical role in encouraging continued participation in sports. However, media professionals tend to be evaluated on their appearance more than their instructional abilities, and prior research on their instructor image is lacking. Additionally, because media professionals often teach exclusively online and cannot take direct responsibility for learners’ outcomes, research on trust in media professionals is limited. Therefore, this study seeks to investigate the relationship between media professionals’ instructor image, trust, and customer satisfaction.

The research hypotheses are as follows:

*H1*: The instructor image of media golf professionals significantly affects instructor trust.

*H2*: The instructor image of media golf professionals significantly affects customer satisfaction.

*H3*: Instructor trust in media golf professionals significantly affects customer satisfaction.

## 2 Materials and methods

### 2.1 Participants

The participants of this study consisted of 300 Korean adult amateur golfers. Data were collected via an online survey conducted between January 7 and March 24, 2025. The participants were recruited using convenience sampling. This study was conducted in accordance with the Declaration of Helsinki. The need for ethical review and approval was waived for this study according to Article 13 of the Enforcement Rule of the Bioethics and Safety Act of the Republic of Korea. To ensure voluntary participation, the following statement was clearly included on the first page of all questionnaires: “Your participation in this study is completely voluntary. There are no foreseeable risks associated with this study. However, if you feel uncomfortable answering any questions, you may withdraw from the survey at any point.” After excluding 10 insincere responses, 290 were included in the final analysis. The demographic characteristics of the participants are presented in Table 1.

**Table 1:** Demographic characteristics of the participants.

Category	Item	*n*	Percentage (%)
Gender	Male	186	64.1
	Female	104	35.9
Age	20s	27	9.3
	30s	81	27.9
	40s	96	33.1
	50 and older	86	29.6
Golf Experience	Less than 1 year	18	6.2
	1–5 years	68	23.4
	5–10 years	83	28.6
	10–20 years	71	24.5
	More than 20 years	50	17.2
Handicap	Under 10	48	16.9
	10–15	54	18.6
	15–20	121	41.7
	Over 20	66	22.8
Viewing Frequency	1–2 times/week	98	33.8
	3–4 times/week	96	33.1
	5–6 times/week	63	21.7
	Daily	33	11.4
Occupation	Office worker	133	45.9
	Professional	48	16.6
	Sales	74	25.5
	Other	35	12.1

### 2.2 Scale

#### 2.2.1 Measurement instruments

To analyze media professionals’ intention to take golf lessons, as well as perceptions of coaching image, trust, and satisfaction, this study utilized and adapted existing validated measurement tools. To measure golf coaching images, this study used and modified an instrument originally developed by Dowling (1986), translated by Park (2012), and later applied in the study of Lee (2017). The instrument was revised to fit the context of this study and consisted of five items measuring qualifications, five items measuring expertise, four items measuring role, and five items measuring attitude. Next, to assess trust in the coach, six items were selected from the questionnaire used by Lee (2010), which was based on Levering’s (1988) measurement tool and adapted and standardized by Cheong (2002). Last, to measure customer satisfaction, this study used a modified version of the questionnaire originally used by Keller (1993) and later adapted by Kim (2000) and Lee (2010). The original instruments were not culturally modified but underwent minor revisions to improve readability in the survey context. To ensure clarity and content validity, the adapted items were reviewed by two faculty members specializing in sport management and three doctoral-level graduate students before data collection. This expert review confirmed the appropriateness and relevance of the items for the target population.

#### 2.2.2 Factor analysis

An exploratory factor analysis (EFA) was conducted to verify the construct validity of the measurement instruments. Factors were extracted by selecting items with eigenvalues greater than 1.0, and items with factor loadings above 0.4 were retained using a repeated estimation of covariance and orthogonal rotation.

Factor analysis revealed that Factor 1 consisted of five items related to expertise, with an eigenvalue of 6.576 and an explanatory power of 34.612%. Factor 2 consisted of five items pertaining to qualifications, with an eigenvalue of 3.024 and an explanatory power of 15.916%. Factor 3 was composed of five items related to attitude, with an eigenvalue of 2.217 and an explanatory power of 11.669%. Factor 4 included four items for the role factor with an eigenvalue of 1.693 and an explanatory power of 8.908%. Thus, the results of the exploratory factor analysis for golf coaching images showed that all factors had factor loadings above 0.5, and the cumulative variance explained was 70.106%, indicating that the survey items had relatively good validity (Table 2). For constructs that have been repeatedly validated as single-factor scales (trust in golf instructor and customer satisfaction) in prior studies, exploratory factor analysis (EFA) was initially considered. However, given their well-established unidimensionality, detailed EFA results were omitted from the final manuscript.

**Table 2:** Results of exploratory factor analysis on instructor image.

Item	Factor 1	Factor 2	Factor 3	Factor 4
Expertise 1	**0.874**	0.142	0.077	0.057
Expertise 2	**0.859**	0.149	0.043	0.081
Expertise 3	**0.850**	0.196	0.088	0.104
Expertise 4	**0.855**	0.185	0.083	0.106
Expertise 5	**0.710**	0.238	0.013	0.272
Competency 1	0.197	**0.852**	0.022	0.105
Competency 2	0.130	**0.856**	0.105	0.084
Competency 3	0.175	**0.823**	0.124	0.076
Competency 4	0.209	**0.803**	0.124	0.045
Competency 5	0.231	**0.690**	0.094	0.076
Attitude 1	−0.051	−0.157	**0.678**	0.202
Attitude 2	−0.018	0.177	**0.656**	0.100
Attitude 3	0.084	0.029	**0.897**	−0.037
Attitude 4	0.105	0.011	**0.892**	0.011
Attitude 5	0.112	−0.18	**0.867**	0.053
Role 4	0.049	0.085	0.088	**0.848**
Role 3	0.092	0.157	0.071	**0.804**
Role 2	0.212	0.249	0.078	**0.740**
Role 1	0.214	0.252	0.032	**0.700**
Eigenvalue	6.576	3.024	2.217	1.693
Variance (%)	34.612	15.916	11.669	8.908
Cumulative (%)	34.612	50.528	62.197	71.106

Bold values show the factor with the highest factor loading for each item.

#### 2.2.3 Reliability analysis

Cronbach’s *α* coefficient was calculated to verify the reliability of the survey instrument. Detailed results of the reliability analysis are presented in Table 3.

**Table 3:** Results of reliability analysis.

Variable	Subfactor	Cronbach’s *α*
Instructor image	Competency	0.907
	Expertise	0.914
	Role	0.833
	Attitude	0.870
Trust in instructor	Single factor	0.891
Customer satisfaction	Single factor	0.891

If the Cronbach’s α value of a survey instrument is above 0.6, it is generally considered reliable. In this study, the reliability of the questionnaire was found to be high. Specifically, Cronbach’s α values for the instructor image ranged from 0.833 to 0.914, while the values for trust in the instructor and customer satisfaction were both 0.891.

### 2.3 Data analysis method

The data analysis method used in this study was SPSS 18.0. The specific procedures were as follows. First, a frequency analysis was conducted to examine the demographic characteristics of the study participants. Second, exploratory factor analyses and reliability analyses were performed to verify the validity and reliability of the golf instructor image, instructor trust, and customer satisfaction scales. Finally, correlation analyses and multiple regression analyses were conducted to analyze the impact of golf instructor image on instructor trust and customer satisfaction. In addition, the regression analysis included diagnostic tests of the residuals, examining normality, homoscedasticity, and autocorrelation (Table 4).

**Table 4:** Correlation analysis results between variables.

Variable	Attitude	Trust	Role	Lesson participation	Competency	Expertise
Attitude	1					
Trust	0.111	1				
Role	0.192^**^	0.312^**^	1			
Customer Satisfaction	0.231^**^	0.177^**^	0.281^**^	1		
Competency	0.200^**^	0.344^**^	0.460^**^	0.273^**^	1	
Expertise	0.146^*^	0.348^**^	0.360^**^	0.332^**^	0.446^**^	1

## 3 Results

### 3.1 Correlation analysis

As shown in Table 5, the correlation analysis revealed a positive relationship between instructor image, trust, and lesson participation. Among these relationships, the correlation between competency and role was the most significant, with a coefficient of 0.460. The second most significant correlation was between competency and expertise. The correlation coefficients ranged from 0.111 to 0.460, which were smaller than the multicollinearity threshold of 0.80, indicating no multicollinearity issues.

**Table 5:** The effect of instructor image on customer satisfaction.

Variable	*B*	*SE*	*β*	*t*	*p*	Tolerance
Attitude	0.162	0.058	0.156	2.815	0.005	0.945
Role	0.129	0.061	0.133	2.131	0.034	0.75
Competency	0.075	0.061	0.081	1.24	0.216	0.69
Expertise	0.217	0.059	0.226	3.667	<0.001	0.77

B, Unstandardized regression coefficient; SE, Standard error; β, Standardized regression coefficient; *t*, *t*-value; *p*, significance level. ^*^p < 0.05; ^**^p < 0.01; ^***^p < 0.001.

### 3.2 The effect of instructor image on instructor trust

**Figure 1:**
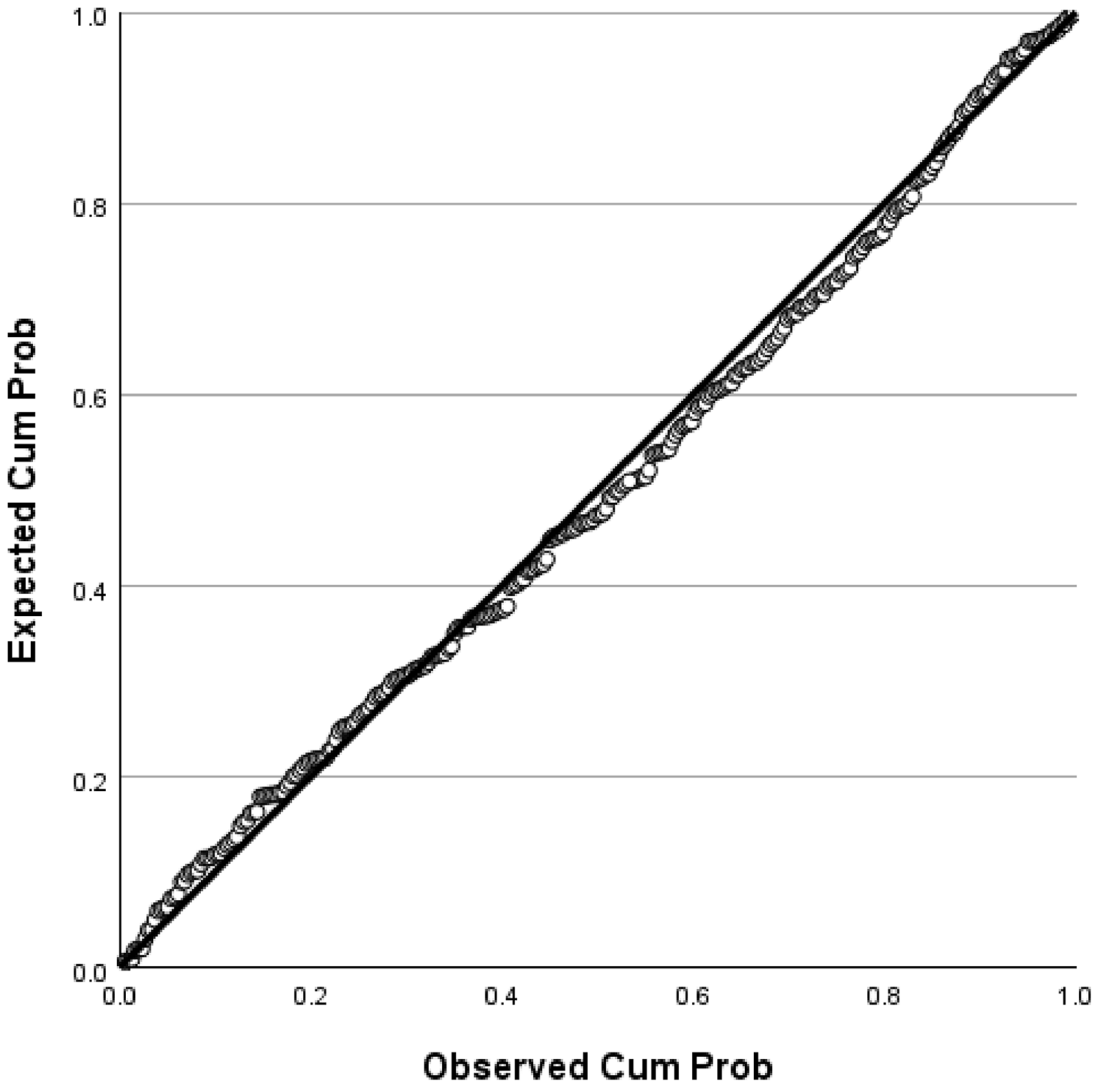
Normal P–P plot of regression standardized residuals.

**Figure 2:**
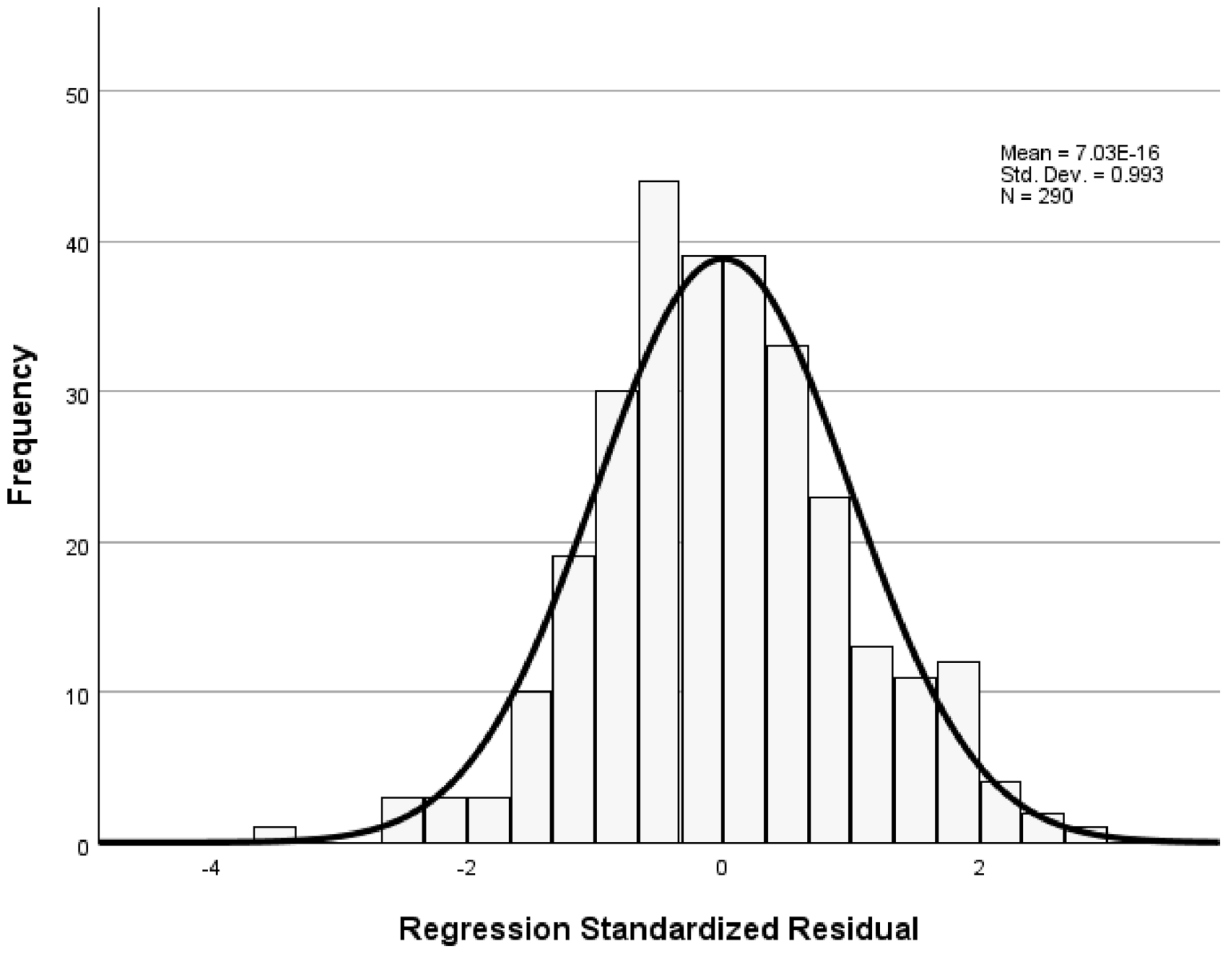
Histogram of standardized residuals.

**Figure 3:**
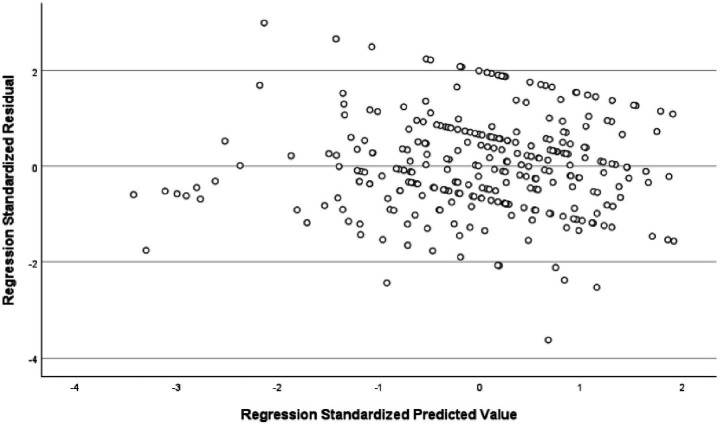
Standardized residuals plot.

The analysis revealed that the model had an explanatory power of 18%, with an *F*-value of 16.029 and a significance level of 0.001, indicating that it was statistically significant. Among the components of instructor image, expertise, competency, and role were found to significantly affect trust in instructors. In contrast, the attitude component showed a t-value of 0.283, which was below the threshold value of 1.96, and a significance level greater than 0.005, indicating that attitude did not have a significant influence on trust in the instructor. All other variables had acceptable t-values and significance levels, and the tolerance values were above 0.1, indicating no multicollinearity. Detailed statistical results were reported in Table 6. The fundamental assumptions of regression analysis were assessed. Normality of residuals was supported, as the standardized residuals in the P–P plot closely followed the diagonal line (Figure 1), and the histogram indicated a mean near zero and a standard deviation close to one (Figure 2). Homoscedasticity was confirmed by the residual scatterplot, which showed a random distribution of residuals around zero without discernible patterns (Figure 3). The Durbin–Watson statistic was 1.854, close to the ideal value of 2, suggesting no autocorrelation. Collectively, these results indicate that the regression model satisfied the assumptions of normality, homoscedasticity, and independence.

**Table 6:** The effect of instructor image on instructor trust.

Variable	*B*	*SE*	*β*		*t*	*p*	Tolerance
Attitude	0.017	0.06	0.016		0.283	0.777	0.945
Role	0.154	0.063	0.151		2.446	0.015	0.95
Competency	0.173	0.063	0.177		2.744	0.006	0.69
Expertise	0.214	0.062	0.212		3.483	<0.001	0.77

### 3.3 The effect of instructor image on customer satisfaction

**Figure 4:**
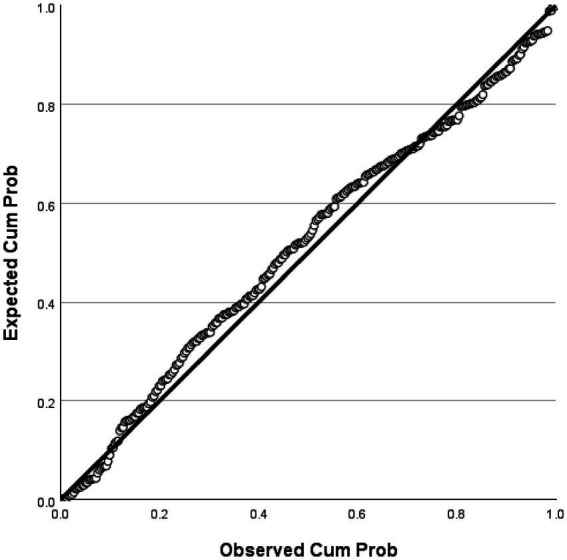
Normal P–P plot of regression standardized residuals.

**Figure 5:**
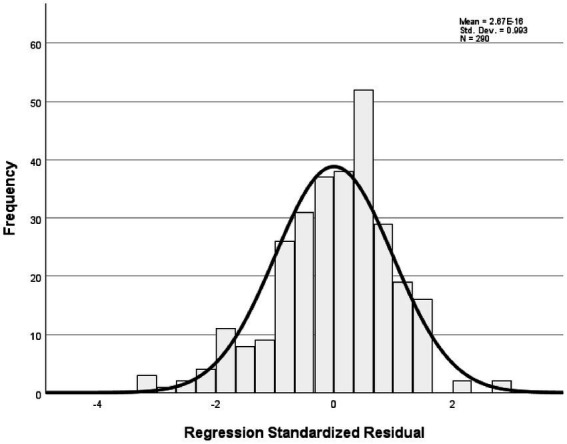
Histogram of standardized residuals.

**Figure 6:**
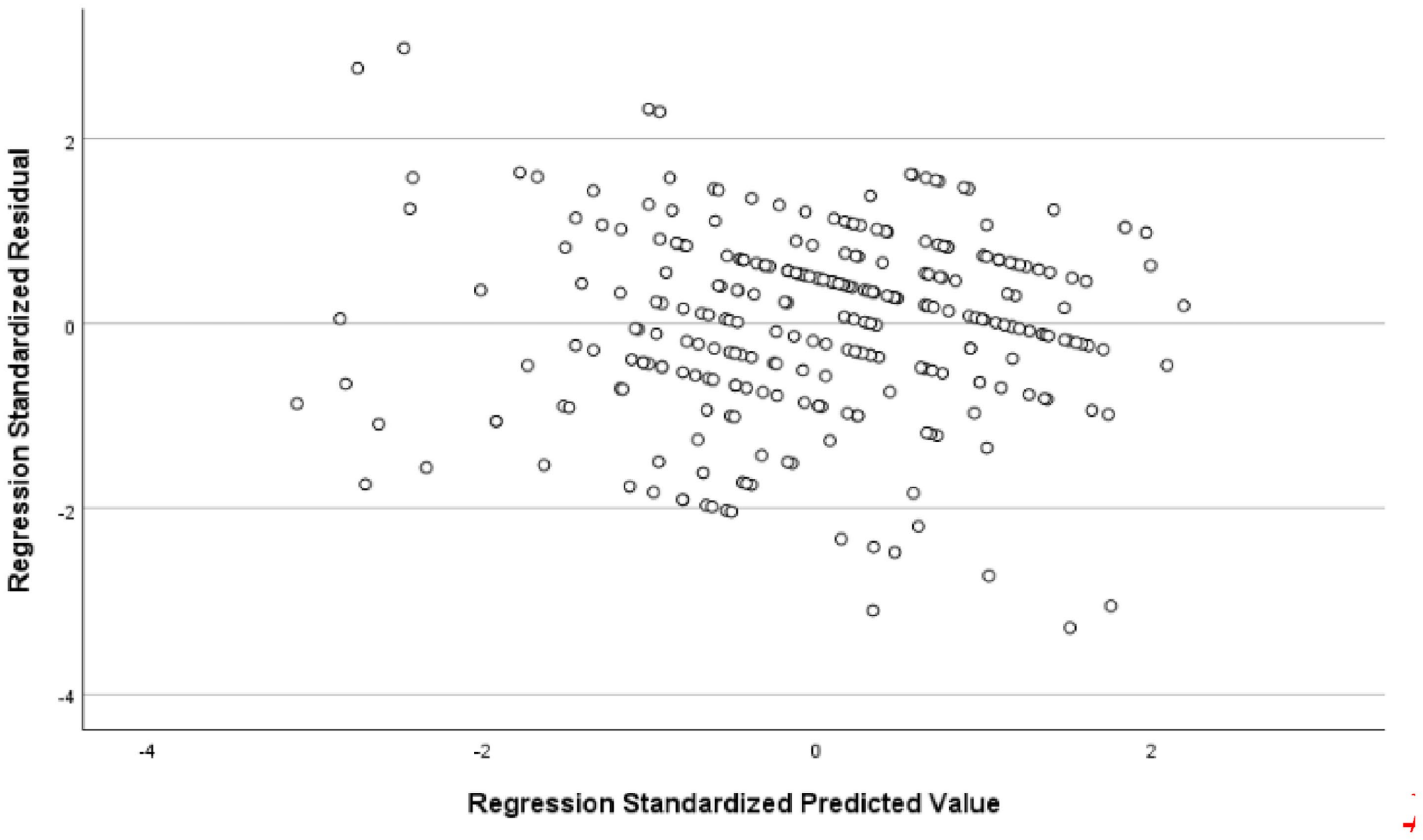
Standardized residuals plot.

The analysis showed that the model had an explanatory power of 17%, indicating statistical significance. The F-value was 14.620 with a significance level of 0.001, suggesting that the regression model was a good fit. Among the components of instructor image, expertise, attitude, and role were found to have a significant influence on customer satisfaction. In contrast, the competency component has a t-value of 1.240, which is below the standard threshold of 1.96, and a significance level greater than 0.005, indicating that competency does not significantly affect customer satisfaction. All other variables met the required t-values and significance thresholds, and the tolerance values were above 0.1, confirming the absence of multicollinearity. Detailed statistical results were shown in Table 5. In this study, the fundamental assumptions of regression analysis were examined. First, the normality of residuals was tested, and the standardized residuals in the normal P–P plot were found to be closely aligned with the diagonal line (Figure 4). In addition, the histogram showed that the mean of the residuals was close to 0 and the standard deviation was close to 1, indicating that the distribution of residuals was generally similar to a normal distribution, thereby suggesting that the normality assumption was not seriously violated (Figure 5). The residual scatterplot further revealed that the residuals were evenly distributed around zero without any specific pattern, supporting the validity of the homoscedasticity assumption (Figure 6). Lastly, the Durbin-Watson statistic was 1.704, which is close to 2, indicating no autocorrelation. Therefore, the regression model in this study was judged to generally satisfy the assumptions of normality, homoscedasticity, and independence.

### 3.4 The effect of instructor trust on customer satisfaction

**Figure 7:**
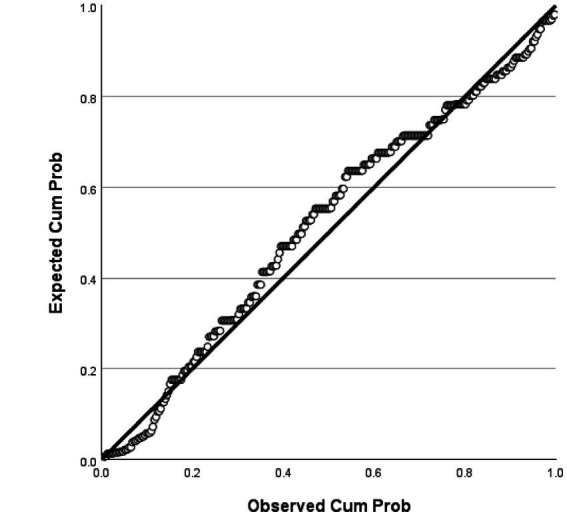
Normal P–P plot of regression standardized residuals.

**Figure 8:**
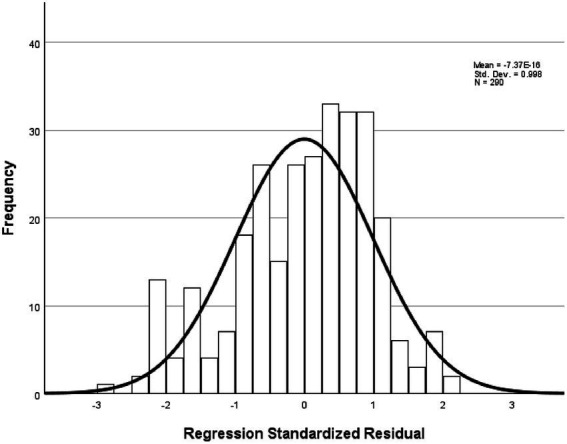
Histogram of standardized residuals.

**Figure 9:**
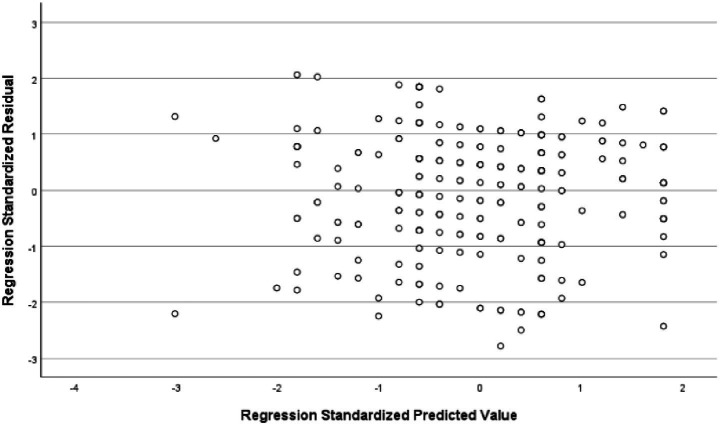
Standardized residuals plot.

Table 7 presents the results of the analysis on the influence of trust in golf instructors on customer satisfaction. The model demonstrates an explanatory power of 3.1%, which is statistically significant. The *F*-value was 9.285, with a significance level of 0.003, indicating that the regression model was appropriate. The t-value was 3.047 and the significance level was 0.003, supporting this hypothesis. Detailed statistical results were reported in Table 7. The fundamental assumptions of regression analysis were evaluated. Normality of residuals was supported by the Kolmogorov–Smirnov test (*p* = 0.040) and Shapiro–Wilk test (*p* = 0.001), as well as by visual inspection of the histogram and normal Q-Q and P–P plots, which indicated distributions consistent with normality (Figures 7, 8). Homoscedasticity was confirmed by the residual scatterplot, which showed a random distribution of residuals around zero without identifiable patterns (Figure 9). The Durbin–Watson statistic was 1.747, close to the ideal value of 2, suggesting no autocorrelation. Collectively, these results indicate that the regression model satisfied the assumptions of normality, homoscedasticity, and independence.

**Table 7:** The effect of instructor trust on customer satisfaction.

Variable	*B*	*SE*	*β*	*t*	*p*
Trust	0.169	0.055	0.177	3.047	0.003

## 4 Discussion

This study examines the image of golf professionals in the media and investigates whether it affects instructors’ trust and customer satisfaction. The results of this study showed that, among the components of instructor image, expertise, competency, and role positively influenced instructor trust, whereas attitude did not show a significant effect, which is consistent with prior findings that professional certification levels strongly shape amateur golfers’ perceptions of expertise, credibility, and participation intentions (Yang et al., 2024). Previous studies have shown that instructors’ or leaders’ attitudes can significantly influence trust and learning outcomes even in non-face-to-face contexts (Schrodt et al., 2009; Li et al., 2021). However, in media-based instruction, attitudes may be less directly perceived by learners, thereby reducing their immediate effect compared to expertise and competence. This difference suggests that the impact of attitudes on trust may vary depending on the instructional environment, underscoring the need for future studies to further examine contextual moderators. Expertise, attitude, and role significantly affected customer satisfaction, whereas competency did not. Finally, instructor trust had a positive effect on customer satisfaction. These results suggest that instructor image positively affects both instructor trust and customer satisfaction, indicating that instructor image plays an important role for learners. Although the explanatory power of instructor image for instructor trust was modest, this finding remains meaningful within the social science context where multiple factors typically shape complex human behavior. It is also acknowledged that unmeasured variables, such as interpersonal communication style, perceived credibility, and prior learning experiences, may further influence the development of instructor trust. Future research should therefore incorporate these additional predictors to achieve a more comprehensive understanding.

Images are formed and shaped by human senses, experiences, and stimuli (Pylyshyn, 1973; Reynolds and Gutman, 1984). An instructor’s image includes competency, expertise, attitudes, and roles. Competency and expertise are objectively evaluated aspects related to an instructor’s ability, whereas attitude and role are emotional aspects that are subjectively evaluated. Instructor expertise refers to outstanding skills and theoretical knowledge in a certain field, and plays an important role in developing learners’ trust (Kao et al., 2017). McAllister (1995) states that learners begin to trust their instructors when they recognize their abilities, implying a close relationship between instructor image and trust. Similarly, Zhang and Chelladurai (2013) suggest that competence, stability, and sincerity are key elements in forming instructor trust. However, attitude did not have a significant effect on the instructors’ trust. Chae et al. (2023) explained that it is difficult to understand another person’s empathy and intentions in non-face-to-face situations. Although some studies have reported that instructors’ attitudes influence trust even in online contexts (Schrodt et al., 2009), other research has shown that expertise and competence in delivering clear information are more critical in forming trust, which aligns with the present findings (Houser and Frymier, 2009; Witt et al., 2004). In face-to-face contexts, direct communication enables learners to more clearly perceive instructors’ attitudes, which has been identified as a key factor in establishing credibility (Li et al., 2021). In contrast, in this study, participants engaged with instructors through media, suggesting that, without the support of specific instructional technologies (Anderson et al., 2001), building trust may be more challenging. Thus, attitudes appear to exert an indirect rather than a direct influence on instructor trust when compared with expertise and competence.

Among the components of instructor image, expertise, attitude, and role positively affected customer satisfaction. In sports, it is important for participants to be satisfied (Burns et al., 2012). Reinboth and Duda (2006) reported that satisfied athletes experienced psychological stability and happiness. Kao and Tsai (2016) state that an instructor’s instructional ability directly affects learner satisfaction, whereas Bendapudi and Berry (1997) report that instructor expertise leads to learner satisfaction. This suggests that when learners evaluate their instructor as having expertise, they feel satisfied with both the instructor and sports performance, a finding that echoes evidence showing that the image attributes of golf star athletes enhance amateur golfers’ participation desire and continuation intention (Yang J. H. et al., 2025). Myers et al. (2011) also found that the technical and competency of the instructor positively affected athlete satisfaction, which partially supports the findings of this study. Eden (2013) stated that learners evaluate an instructor’s competency and that their satisfaction is determined by the interaction. However, competency does not have a significant influence on customer satisfaction. Competency includes enthusiasm (passion), understanding, and the instructor’s ability to positively lead the learner (Myers et al., 2010). However, because the participants in this study did not interact with instructors face-to-face, it is possible that the lack of emotional closeness and sense of security experienced in face-to-face settings influenced the results (Passmore et al., 2023). Nevertheless, both this study and Prentice and Cownie (2020) confirmed that instructor trust can be built even in non-face-to-face situations. Moreover, recent studies on virtual golf and exergaming highlight continuous participation benefits among older adults (Choi et al., 2024), while evidence from super-aging societies demonstrates that health concerns, athletic passion, and leisure satisfaction drive sustained participation (Jun and Choi, 2024). Differences across age groups in enjoyment, exercise commitment, and continuation intention have also been observed (Yang H. J. et al., 2025), extending the implications of the present findings. As situations differ, it is assumed that different elements are involved in building trust. As the results show, learners feel satisfaction depending on the expertise and instructional ability of the instructor; therefore, it is important that instructors continue to develop themselves personally (Irving, 2021). Although non-face-to-face instruction has strengths such as convenience and efficiency (Kinnunen and Georgescu, 2020), to ensure continued viewing and engagement, instructors must put in effort to enhance their instructional competence.

Trust in instructors has a significant effect on customer satisfaction. Bandura and Kavussanu (2018) stated that higher instructor trust increases sports satisfaction, and that trustworthy instructors are authentic and provide autonomy. Trust is strengthened when an instructor is genuinely committed to a learner’s development and provides clear goals and motivation (Gulak-Lipka, 2017). LaVoi (2007) emphasizes that when an instructor understands and respects the learner, the learner feels close and tries to maintain the relationship. This promotes positive social interaction, and learners experience satisfaction through this process. Li et al. (2021) also reported that high instructor trust reduces learners’ anxiety. Despite different backgrounds, this study found a positive relationship between instructor trust and customer satisfaction. Jowett (2017) stated that when learners trust their instructors, they achieve better results through collaborative relationships. This suggests that when trust is built into the instructor, learners feel satisfied and are positively influenced by their skill development.

Taken together, this study confirms that even media golf professionals can form a positive instructor image, and that such an image can contribute to building instructor trust. Expertise and instructional ability are the most important factors in forming an instructor’s perception and trust. Because golf professionals are mostly encountered through the media or online, factors such as attitude, including sincerity and confidence, were not found to influence instructor trust or satisfaction. These results show that instructor image, instructor trust, and customer satisfaction are significantly related. When an instructor’s image is well established, trust is built, which leads to customer satisfaction–a connection confirmed in this study.

### 4.1 Conclusion

This study aims to empirically analyze the impact of media images of golf professionals’ instructors on trust and customer satisfaction. One of the major findings of this study was that media professionals can maintain a positive instructor image, which can effectively build instructor trust. The results indicated a strong relationship between instructor image and both instructor trust and satisfaction. As demonstrated in this study, media professionals who possess the right qualities as instructors can use their instructor’s image to gain learners’ trust, leading to higher satisfaction. The most crucial elements in building an instructor’s image and trust are expertise and instructional ability. Because golf professionals are often encountered through media or online platforms rather than face-to-face, factors such as sincerity and confidence were not found to significantly affect instructors’ trust or satisfaction. Therefore, it is essential for media golf professionals to focus not only on their external appearance, but also on their expertise and instructional abilities. Even in online environments, when instruction is provided through a systematic educational framework, learners can access reliable information and enhance their learning satisfaction (Picciano, 2002). Therefore, by designing and implementing a well-structured educational system, learners are likely to continue engaging with the instructional content. The current generation communicates and gathers information through media (Amedie, 2015); thus, there is likely to be a high demand from this audience. Learners feel satisfied when media professionals deliver accurate information and demonstrate expertise as instructors (Fouraki et al., 2020). Media professionals should continue to improve in order to meet these demands. Several studies have indicated that when instructors engage in prosocial communication, they foster greater trust (Ellis, 2000; Frymier and Thompson, 1992; Schrodt et al., 2009). Therefore, instructors should focus on improving learners’ skills and engagement, actively accepting feedback and pursuing continuous improvement (Schrodt et al., 2006). Additionally, nonverbal behaviors can make learners feel a closer connection and greater satisfaction (Baker, 2010; Finn et al., 2009; Wu et al., 2022), suggesting that instructors should leverage these behaviors to clearly establish their presence. Now, as they have become a distinct profession, to maintain their continuous popularity, they need to focus on developing their instructional expertise and, even in non-face-to-face situations, ensure that they provide authenticity and effective guidance to their learners.

### 4.2 Study limitations and future directions

Based on the results of this study, the following suggestions are made: First, it focused on media professionals; therefore, it did not identify any differences with professionals who provided offline lessons. Future research should categorize and analyze both groups in-depth to explore their differences and ways of enhancing their effectiveness as instructors. Second, it is difficult to assess instructor images and perceptions accurately using data from a single group. It would be beneficial to conduct surveys across multiple groups over different periods to gain deeper insights into the image of media professionals as instructors and provide a more comprehensive analysis. Third, the cross-sectional design with a convenience sample of Korean amateur golfers inevitably limits the generalizability of the findings. Nevertheless, the sample size was statistically sufficient, and participants were drawn from diverse demographic and golfing backgrounds to mitigate bias. Future research employing probability sampling and longitudinal designs is recommended to enhance the robustness of the results. Last, Construct validity was assessed through exploratory factor analysis (EFA), which showed acceptable loadings, eigenvalues, and explained variance. However, the absence of confirmatory factor analysis (CFA) is recognized as a limitation, and future studies should employ CFA to further validate the factor structure.

## Data Availability

The original contributions presented in the study are included in the article/supplementary material, further inquiries can be directed to the corresponding author/s.
